# A Perspective on Lung Cancer and Lung Microbiome: Insight on Immunity

**DOI:** 10.1002/iid3.70145

**Published:** 2025-01-31

**Authors:** Reza Emadi, Sasan Saki, Parastoo Yousefi, Alireza Tabibzadeh

**Affiliations:** ^1^ Department of Medical Laboratory Sciences, Faculty of Medical Sciences Islamic Azad University, Arak Branch Arak Iran; ^2^ Department of Virology, School of Medicine Iran University of Medical Sciences Tehran Iran; ^3^ Applied Neuroscience Research Center Islamic Azad University, Arak Branch Arak Iran

**Keywords:** carcinogenesis, lung cancer, microbiome, microbiota

## Abstract

**Background:**

Although the carcinogenic potential of microbes has long been recognized, their significance may have been underestimated. Currently, the connection between microbiota and cancer is under extensive research. The lung microbiota may serve as a proxy for the state of lung health based on its crucial role in preserving lung hemostasis.

**Objectives:**

This review tried to outline the state of our understanding of the contribution of lung microbiome and lung cancer.

**Methods:**

A literature search was performed using PubMed, Google Scholar, and Scopus databases for recent research focusing on the development and possible pathogenesis of lung microbiome and lung cancer.

**Results:**

Early research on lung cancer indicated that dysbiosis significantly impacted the development and spread of the tumor. As a result of these findings, the study of the lung microbiota as a possible therapeutic target and diagnostic marker has accelerated. Early‐stage disease diagnostic biomarkers could be represented as microbiota profiles. Additionally, the microbiome is involved in anticancer therapy. There are limited studies on lung microbiota, and most microbiome studies commonly concentrate on the gut microbiota. A proper understanding of lung microbiota can have several potential therapeutic approaches. Therefore, more studies in this field may initiate remarkable advancements in microbiome‐dependent treatment.

**Conclusion:**

Convincing data from studies on both humans and animals indicates that the microbiota might play a role in cancer initiation, influenced by internal and environmental factors of the host. Notably, the lung harbors its microbiome, as do lung cancers. In general view, it seems microbiome diversity in lung cancer patients is reduced. Meanwhile, some genera were increased in lung cancer patients in comparison with a noncancerous population (such as *Streptococcus* genus), and some of them were decreased (*Granulicatella adiacens*, *G. adiacens*). Furthermore, research on the microbiome‐carcinogenesis relationship is still in its infancy, and much remains to be fully understood.

## Graphical abstract

Although the carcinogenic potential of microbes has long been recognized, their significance may have been underestimated. This review outlines the state of our understanding of the contribution of microbiomes, particularly lung microbiomes, to lung cancer.

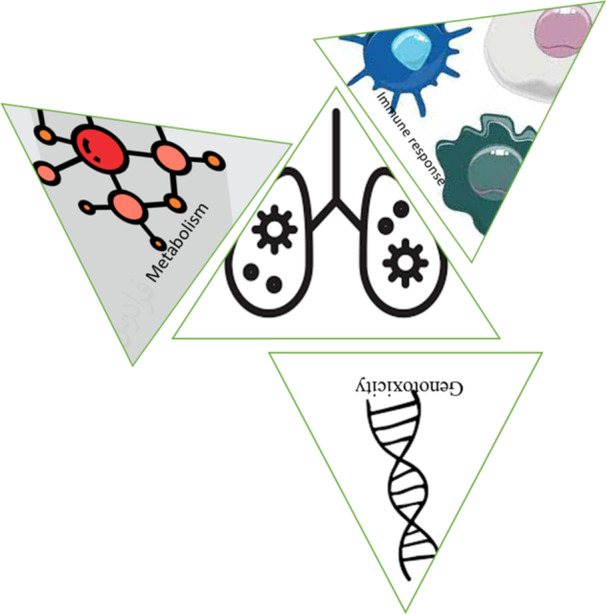

## 1 Introduction

The International Agency for Cancer Research states that at least 10 distinct biological agents, including bacteria, viruses, and parasites, have been linked to the etiology of cancer [1]. The microbiome's count and diversity differ from anatomical sites and are influenced by environmental and host variables that can play a role in disease and health [2]. Microbiota is a component of the innate immune system and regulates many host defense mechanisms [3]. The rates of immigration, elimination, and replication of the microorganisms influence the composition of this microbiota [4].

Initially, epidemiological research on individuals could show the connection between microbes and cancer. According to certain research, bacteria have a part in the malignant transformation of mucosal cells. Studies on the carcinogenicity of *Fusobacterium nucelatum* (*F*. *nucleatum*), a bacterium in the mouth cavity known to cause periodontal disease. It has been demonstrated that this bacterium is associated with colorectal cancer lesions [5, 6]. Additionally, it has been linked to a poor prognosis of colorectal cancer which may be helpful for prevention and treatment [7]. The link between gut microbiota and gastrointestinal and metabolic disorders, including gastric cancer, is the most well‐studied microbiome‐cancer relationship [8]. Since the gut is the primary site of colonization of commensal bacteria, the link between gut‐related pathogenesis and microbiota is not unbelievable. However, recently, it has also been discovered that other healthy tissues and organs, such as the lower respiratory tract, can harbor a mass population of microbes. As a result, research on respiratory microbiology concepts is ongoing [9]. It was previously thought that the lung was a Taxa‐free environment, but in actuality, the lungs are host to a colony of bacteria that is primarily composed of *Firmicutes*, *Proteobacteria*, *Actinobacteria*, and *Bacteroidetes* [4].

Lung cancer is one of the primary contributors to cancer‐related fatalities worldwide, affecting all genders [10]. Tobacco consumption [11], genetic predisposition [12], and viral infections such as EBV [13], have important roles in the progression of lung cancer. Forecasting the risk of cancer growth and enhancing treatment efficacy and safety may depend on our ability to comprehend how microorganisms are found in the respiratory tract. This can impact lung cancer development and treatment efficacy.

In this current review, we attempted to present an overview of the scientific state to enhance our understanding of how the composition and function of the lung microbiome might influence the development of lung cancer. We discuss the importance and role of the lung microbiome in healthy individuals, patients with non‐cancer diseases, and the function of the lung microbiome in patients with lung cancer.

## 2 General Concepts of Lung Microbiome in Noncancerous Individuals

*Fusobacteria Firmicutes, Proteobacteria*, and *Bacteroidetes* are the principal phyla and classes in the healthy respiratory tract, whereas the principal flora genera include *Veillonella, Pseudomonas*, *Haemophilus, Streptococcus*, *Prevotella*, and *Neisseria* [14, 15]. The primary source of the lung microbiome in healthy individuals appears to be the micro‐aspiration of pharyngeal secretions [16, 17, 18]. These genera may not be found in all healthy individuals, though, as research has shown that the lung microbiome can be divided into two groups known as pneumotypes based on the taxonomy and bacterial load. Known as the supraglottic predominant taxa (SPT) pneumotype, the initial group is enriched with number of bacteria and is particularly abundant in oral microorganisms, include *Veillonella* and *Prevotella*. The second group, referred to as the background predominant taxa (BPT) pneumotype, is characterized by a minimal presence of bacteria and includes environmental taxa like *Pseudomonas* and *Acidocella* [19].

**Figure 1:**
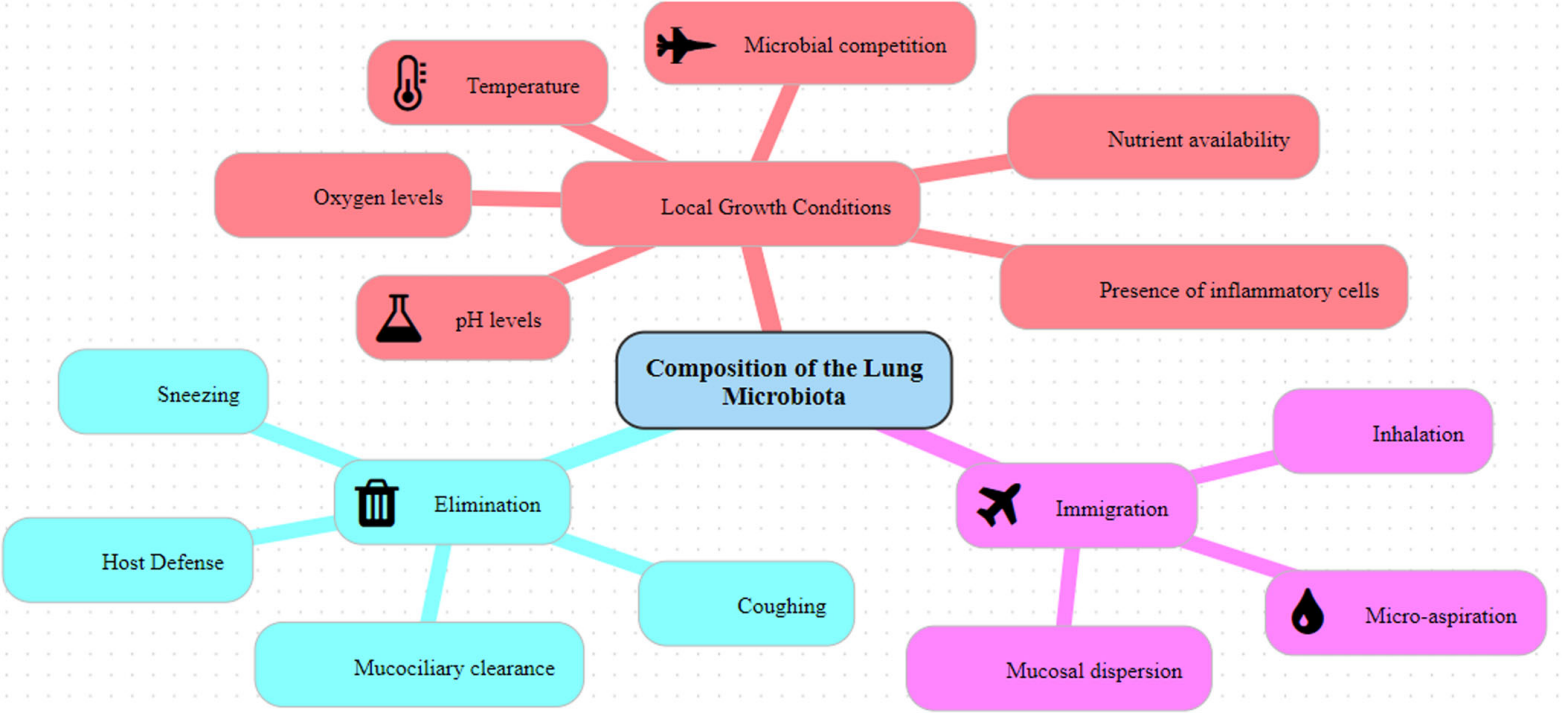
Local growth factors, elimination, and immigration are the three elements that influence the dynamics of the host microbiota constitution in the lungs.

Three ecological processes influence the composition of the microbiota; Immigration, elimination, and the impact of local growth circumstances on the community members’ replication rates. Disturbances in one or more of these variables can reveal changes in the microbiota and cause Dysbiosis [4]. The term dysbiosis refers to any alteration in the composition of the resident commensal community [20]. The primary mechanisms controlling the composition of the microbiota in the lung are the localized growth circumstances that impact replication rates [4] (Figure 1).

Changes in local growth conditions lead to dysbiosis, which modifies the population of commensal microorganisms [21]. If the irritating stimulus is eliminated, this alteration can be reversed; but, if sufficient inflammatory harm to the epithelial cells has been done, it cannot be reversed [22, 23]. Dysbiosis leads to airway inflammation and Elevated mucus production and permeability are caused by airway inflammation, which enhances the bacterial burden by producing regions of elevated temperature and hypoxia, as well as by increasing the amount of nutrients that reach the lungs [21]. The etiology of long‐term lung conditions, including asthma, cystic fibrosis, idiopathic pulmonary fibrosis, and chronic obstructive pulmonary disease (COPD), has been linked to the microbiome [24]. Consistent with these discoveries, Dicker et al. discovered that worse COPD characteristics and more frequent exacerbations were linked to decreased microbial diversity and *Proteobacteria* dominancy [25].

In patients with acute lung infections, including pneumonia, investigation of the composition of the microbial population has not been thoroughly investigated, possibly because of the limited duration of these diseases. But utilizing Germ‐Free (GF) mice models in various situations, it has been shown that the host microbiota plays an advantageous function during acute bacterial infections of the lungs. For instance, GF mice are more susceptible to lung infections caused by *Streptococcus pneumoniae, Klebsiella pneumoniae*, and *Pseudomonas aeruginosa* [26, 27]. Schuijt et al., have induced dysbiosis in mice and evaluated the contribution of the bacterial microbiome to resistance to lung infections using combinations of broad‐spectrum antibiotics. They have demonstrated that Antibiotic‐treated mice are more sensitive to respiratory pathogens such as *S. pneumoniae* and *K. pneumonia*. Specifically, antibiotic‐treated mice infected with *S. pneumoniae* exhibited a deficiency in lung cytokine production [28]. Brown et al. have also shown, the vulnerability of antibiotic‐treated mice to *S. pneumoniae* and *K. pneumoniae* infection was connected with decreased lung production of interleukin (IL)‐17A and granulocyte‐macrophage colony‐stimulating factor (GM‐CSF) [26].

## 3 Lung Microbiome and Lung Cancer

Lung cancer is a multifactorial process that includes dynamic alterations in the respiratory microbial structure. Consequently, it is vital for study and useful for utilization when the bacterial structure in the respiratory tract is improved, as this may have the ability to control the development of cancer [29]. According to McLean et al., dysbiosis may cause cancer through three different mechanisms: impaired immune balance, induction of oncogenic pathways, and chronic inflammation [21]. Initially, dysbiosis has the potential to interfere with the lungs' natural immune system stimulation. The priming of antigen‐presenting cells is hampered by the loss of microbial diversity, which limits the cells' capacity to respond to tumor antigens [22]. Furthermore, lung cancer is associated with the microbiome, and the microbiome affects on immune system especially helper T cells and the inflammation process [30]. Secondly, the release of genotoxins and DNA‐damaging compounds by the impacted commensal organisms results in chronic inflammation caused by dysbiosis [31]. Ultimately, much research discovered that some microbiome species may directly trigger oncogenic pathways (Table 1).

**Table 1:** Bacterial communities found in patients with lung cancer (some of dominant studies mentioned).

Bacteria	Sample		Method^a^	Ref.
	Size	Type		
Granulicatella	16	Sputum	V1 and V2	[32]
Abiotrophia				
Streptococcus				
Streptococcus	40	Lung tissue	V4	[33]
Prevotella				
Veillonella	28	BAL	V1‐V3	[34]
Megasphaera				
Cyanobacteria	29	Lung tissue	—	[35]
Acidovorax	176	Lung tissue	V3‐V5	[36]
Veillonell	40	BAL	V3 and V4	[37]
Megasphaera				
Actinomyces				
Arthrobacter				
Rothia				
Streptococcus				
Veillonella				
Capnocytophaga				
Prevotella	91	BAL	—	[38]
Bradyrhizobium japonicum				
Streptococcus	85	Airway brushes	V4	[39]
Viellonella				
Capnocytophaga	30	saliva	V3 and V6	[40]
Veillonella				

Abbreviation: BAL, bronchoalveolar lavage.

^a^ The studies conducted to investigate the microbiota have usually been performed utilizing the 16S rRNA gene sequencing method. The nine hypervariable regions (V1–V9) of the bacterial 16S rRNA gene, which range in length from 30 to 100 base pairs, are involved in the secondary structure of the ribosomal small subunit [41].

Liu, H.X. et al. profiled the lower airway microbiome of 24 lung cancer patients with unilateral lobar masses and 18 healthy controls using samples from protected bronchial specimens. The microbiome diversity was significantly lower in cancer patients compared to controls and steadily declined from healthy to noncancerous to cancerous sites. In cancer cases, the presence of the *Streptococcus* genus was notably greater, whereas controls exhibited elevated levels of *Staphylococcus*. This also suggests microbial changes may be involved in lung cancer development [42].

Cameron and colleagues analyzed the potential of using the sputum microbiome as a source of noninvasive bacterial markers to determine lung cancer status and stage. Analysis found some bacterial species were present at significantly higher abundances in lung cancer‐positive samples, including *Granulicatella adiacens* and *G*. *adiacens* [43].

According to Prior research has demonstrated that individuals with lung cancer typically have altered bacterial composition, decreased overall bacterial abundance, and a lack of diversity in their microbiome [34, 38, 44]. However, it is also true that different have depicted varying concentrations of bacterial genera in individuals with lung cancer. Variations in the outcomes between samples show the dynamic involvement of microbiota in carcinogenesis, implying that certain taxa may have a unique function at various stages and host locations [45]. It's crucial to remember that it might be challenging to determine whether respiratory disorders are caused by the microbiome. Also, hard to identify whether the source of a bacterial colony in the respiratory tract is from an endogenous bacterium, or due to a decline in airway resistance, infections, or other potential sources [15]. Tsay et al. [39] investigated the role of the host microbiome in cancer pathways in lung cancer samples. The study indicates that ERK (extracellular signal‐regulated kinase) and PI3K (phosphoinositide 3‐kinase) signaling pathways were upregulated in cancerous samples in comparison with controls. These upregulation were due to an increased number of oral taxa such as *Streptococcus, Prevotella*, and *Veillonella* in lower respiratory tract samples.

On the other hand, it is the notion of the gut‐lung axis [46]. Recent studies have demonstrated that immune homeostasis maintenance and disease pathways require bidirectional contact between the gut and the lung [47]. Surprisingly, research has shown that the gut microbiota affects lung function through the “gut‐lung axis,” a vital channel for communication between the gut microbiota and the lungs [48, 49]. The gut‐lung axis is thought to be a two‐way interaction, implying that microbial metabolites and endotoxins may influence the lung through blood, and when inflammation arises in the lung, the lung can also influence the gut microbiota [50].

It should be mentioned that various research indicates that the majority of bacterial DNA found in the lungs may originate from bacteria that are not viable. In fact, it was discovered that over 90% of microbial DNA was DNase I sensitive, suggesting that the source of this DNA was nonviable microorganisms [51, 52]. Additionally, Willis et al. discovered that nonviable sources accounted for up to 50% of the bacterial DNA in sinus tissue [52]. Contrary to this finding, independent investigations have revealed that, with the proper procedures, the microbes found in the lower respiratory tract may be cultivated, proving that this microbiome comprises more than just bacterial DNA or debris [53, 54]. It suggests a potent area of research for future studies in the way of more clarification of the role of the microbiome in lung tissue and the most trustable method for evaluating it.

Regardless of lung cancer development and progression, the lung microbiota is related to lung cancer metastasis and histology of the tumor. It's been suggested that Veillonell, Megasphaera, Actinomyces, and Arthrobacter genera were more frequent in adenocarcinoma lung cancer type in comparison with squamous cell lung carcinoma. Also, adenocarcinoma lung cancer with metastasis represents less Streptococcus genus in the evaluated sample in comparison with non‐metastasis patients [37]. While this association of microbiota with lung cancer was suggested in other studies [55], these findings need more evidence for a clear conclusion.

## 4 Microbiota‐Related Pathways in the Lung Cancer

Alteration in lung microbiota can be a start for other metabolic and toxic pathways in the way of tumorigenesis. Determining the function of microbiota in lung cancer formation and the mechanisms by which bacteria regulate tumor growth or progression are crucial steps to provide a deeper understanding of lung cancer and microbiota association. Most experts agree that cancer is a complex disease caused by abnormal cells proliferating uncontrollably. This involves disruptions to autophagy and apoptosis, which leads to inflammation and damage to DNA [56, 57]. Bacterial‐produced genotoxins and metabolites cause direct harm to the host's DNA, triggering genomic instability through reactive oxygen or nitrogen species, as well as natural killer immune receptors [58]. This process results in cancer‐related changes when the combined damage surpasses the host's capacity for self‐healing [58]. Genotoxic *Escherichia coli* with a pks gene (pks+ *E. coli*) injected organoids showed a distinct mutation profile in a recent study, indicating direct bacterial participation in the occurrence of carcinogenic mutations [59]. Generally, three main mechanisms have been shown as possible mechanisms of action that are considered one by one below including genotoxicity, metabolism, and immune response.

### 4.1 Microbial Metabolites and Metabolism

Several bacterial metabolites affect the host's signaling pathways and metabolism regulation. Therefore, alterations in bacterial‐generated substances arising from biological irregularities within the tumor microenvironment may impact lung cancer cells' oncogenic signaling and metabolism [60]. The composition and structure of bacterial metabolites are more complex than host metabolites, and they can influence the differentiation tendency of T regs, naïve T cells, effector T cells, or the release of T helper type 17 (Th17), which can initiate systemic inflammation and the immune response. The investigation of intestinal dysregulation of host metabolism through modifications to the microbiome has been ongoing [61]. Tumor development may be aided by disturbance of the metabolic balance brought on by the microbiome's altered homeostasis. Moreover, it has been suggested that the production of carcinogenic metabolites initiates this process by modifying the host's inflammatory response and suppressing immunological responses. Consequently, research on the gut microbiome's metabolome may be helpful in the identification of cancer. The main cause of this is the development of high‐throughput techniques, which have transformed genetic and molecular research and led to the identification of several cancer biomarkers and other extremely intricate interspecies interactions. The range of compounds generated by intestinal bacteria penetrates the circulatory system and controls the physiopathological state of other organs, such as the lungs. For instance, the gut‐lung axis, which controls how the lungs react to substances from intestinal bacteria in peripheral circulation [62]. Short‐chain fatty acids (SCFAs) are among the vital metabolites abundantly generated by commensal microorganisms, serving pivotal roles in molecular signaling pathways. Extensive research has delved into their impact on the gut and host immune system, yet their influence on the respiratory system, epithelium, and immune responses remains largely unexplored. Notably, a recent investigation unveiled that fermentable dietary fiber like inulin can reshape gut bacteria composition and associated metabolites, including SCFAs. This alteration was linked to enhanced mouse response against influenza virus infection, attributed to reduced neutrophil‐induced damage and boosted the CD8^+^ T‐cell antiviral reactions. Moreover, the biofilm enveloping the lower respiratory tract's epithelium creates an ideal hypoxic environment, facilitating anaerobic bacteria's fermentation process for energy acquisition. This biofilm is notably rich in SCFAs, which serve as intermediate products in the anaerobic metabolism of microorganisms [63]. Elevated lung SCFA levels may cause effector T cell fatigue, reduce cytotoxicity to cancerous cells, and prevent CD4^+^ and, CD8^+^ T cells from producing IFN‐γ [64]. Numerous studies indicate certain bacteria contribute to the production of acetaldehyde, a well‐known carcinogen. Conversely, deoxycholic acid (DCA), an obesity‐induced metabolite originating from the gut microbiome, is implicated in the development of hepatocellular carcinoma associated with obesity. Moreover, DCA may facilitate the activation of histone deacetylase‐like 3 (HDAC3) via inositol triphosphate pathways, thereby influencing intestinal homeostasis and supporting repair mechanisms [65, 66].

### 4.2 Genotoxicity and Toxins

DNA damage to a host can alter the expression of oncogenes and tumor suppressor genes, as well as result in cell death. It has been demonstrated that genotoxicity is associated with certain bacterial molecules. For instance, it has been demonstrated that the toxins *Bacteroides fragilis* and *E. coli* are implicated in the generation of double‐stranded DNA damage [67, 68]. Bacteria generate chemical substances, including the active oxygen species from *Porphyromonas*, hydrogen sulfide from *Clostridium cholephilum*, and superoxide dismutase from different bacterial strains. These substances contribute to genomic instability, thereby increasing the likelihood of cancer occurrence. Imbalances or alterations in the bacteriome's composition can result in the production of toxins that prompt the release of free radicals by histiocytes or bacteria. These radicals, in turn, exert a carcinogenic effect on the host organism. Disruptions in bacterial balance, whether due to aging or exposure to xenobiotics, may induce the production of antimicrobials or a mixture of toxins aimed at competing rivals [30, 69]. Additionally, a cytotoxic distension toxin (CDT) generated by several Gram‐negative bacteria including *E. coli*, *Haemophilus ducreyi*, *Helicobacter* sp., *Shigella dysenteriae*, and *Campylobacter jejuni*, and typhoid toxin (TT) produced by *Salmonella enterica* serovar Typhi directly affect the DNA integrity of host biological target cells [70].

Greathouse et al. proposed a hypothesis concerning the interaction among smoking, TP53 mutations, and the microbiota in smoking‐induced lung carcinogenesis. They suggested that lung epithelial cells carrying TP53 mutations caused by tobacco smoke could create a conducive microenvironment that promotes the invasion of certain bacterial species. These bacteria, thriving in the altered environment, may potentially act as promoters in the process of lung tumorigenesis [71].

Certain lung bacteria have the capability of transferring plasmids containing genetic traits to tumor cells using exosomes. This transfer can lead to carcinogenic effects, including drug resistance and pro‐inflammatory responses. Additionally, deoxycholic acid and lithocholic acid, which are secondary metabolites produced by intestinal bacteria from bile acids, have been implicated in DNA damage and initiating cancer. The metabolic imbalance in the lungs can result in the formation of detrimental metabolites, which may further contribute to the development of lung cancer [66].

### 4.3 Immune Response

One of the possible pathogenesis pathways of lung cancer is disruption in the microbiota, which plays a critical role in maintaining the balance between inflammation and antitumor immunity [67]. The microbiome has a significant impact on how adaptive immunity develops throughout a person's lifespan. The lung microbiome influences the immune system by adjusting the host's susceptibility to various pathogenic causes and treatment results. On the other hand, the host immune system and outside factors modify and maintain the state of the microbiome. The growth of pathogenic bacteria is maintained, and the populations of common bacterial species are kept in balance [66]. Therefore, it is conceivable that modifications to the microbiome might control the host's immunological response. Thus, a thorough comprehension of the inflammatory pathways regulated by the bacteria and the immune response is necessary.

After Toll‐like receptors (TLRs) identify the microbes, NF‐κB and STAT3 are triggered, which results in the differentiation of Th17 cells. Therefore, excessive stimulation of the mucosal immune system because of bacterial overgrowth can also result in an infinite activation of inflammatory Th17 cells, which promote inflammatory and carcinogenic pathways [30, 66, 68]. A new study looked at how the immune system's defense against lung cancer is affected by the gut microbiome. In mice, enterotoxigenic *B. fragilis* (ETBF) triggers a specific Th17 response that activates STAT3, indicating that human commensal bacteria may contribute to cancer via a Th17‐dependent mechanism [70]. The lung bacteria also regulate the production of innate immunity genes, such as IL‐5, IL‐10, and IFN. In the lungs of SPF (specific pathogen‐free) neonates compared to GF (germ‐free) mice, the expression of PD‐L1 on CD11_C_ DCs and FoxP3^+^, CD25^+^ Treg cells was enhanced [71]. GF mice without intestinal flora showed severe immunological defects in preclinical investigations, including an insufficient mucous layer, an anomaly in the generation of immunoglobulins, and a decrease in the size and number of lymph nodes [72].

Compared to healthy controls, patients with NSCLC respond to *Streptococcus salivarius* and *Streptococcus agalactiae* with considerably more Th17 cells and elevated Th1 cells [73]. It has recently been demonstrated that *Pasteurella* has a negative association with M2 macrophages and a favorable correlation with cytotoxic CD8^+^ tumor‐infiltrating lymphocytes (TILs). Additionally, it has been demonstrated that *Coriobacteriaceae* and M2 macrophages have a substantial positive association, while *Coriobacteriaceae* and CD8^+^ TILs have a negative correlation [74]. The Th17 inflammatory phenotype may be an additional mechanism, apart from the Th2 response, in patients with lung disease. IL‐17 overexpression and the Th17 inflammatory pathway in particular may not be the sole increased individuals in a host with lung dysbiosis. According to a recent study, pathogen penetration into the epithelium in polymeric Ig receptor (pIgR) deficient mice is caused by a lack of mucosal immunity, which intensifies the inflammatory response and increases lung infection caused by bacteria. Furthermore, under conditions like persistent inflammation, bacteria that are unable to survive by metabolizing inflammatory chemicals may be out‐competed by *Gammaproteobacteria*. *Gammaproteobacteria* may use reactive nitrogen species, which are produced by a lot of inflammatory cells, as a terminal acceptor for electrons to continue growing in an inflammatory environment. Thus, it is plausible that the changed microbiome brought about by potentially hazardous microorganisms would be encouraged [75, 76].

Cheng et al. showed an inadequate induction of lung immunity following antibiotic therapy, highlighting the significance of commensal bacteria in bolstering the host immune response against cancer [77]. According to Le et al., the modification of the immune response is linked to the reduction in the implantation of experimental lung metastases following local antibiotic therapy [78]. These findings show the concern of commensal bacteria in preserving the host's immunological homeostasis.

## 5 Microbiota and Lung Cancer Treatment

Despite improvement in the treatment of lung cancer, unfortunately, a significant number of patients suffer from delays in diagnosis, which makes diagnosis with advanced‐stage disease. Consequently, it's critical to determine early diagnosis techniques and optimize the treatment for lung cancer [79]. Recognizing the relationship between lung and gut microbiota and lung cancer might change the lung cancer treatment effectively.

Monoclonal antibodies, including those that inhibit programmed death 1 (PD‐1), programmed death‐ligand 1 (PD‐L1), and cytotoxic T lymphocyte‐associated protein 4 (CTLA‐4) are often employed in cancer immunotherapy, especially for advanced NSC lung cancer. These immune checkpoint blockers (ICBs) have shown significant development potential in cancer treatment in recent years. Two targets that have received a great deal of interest are PD‐L1 and CTLA‐4 [80, 81, 82]. There have also been reports on the connection between Bifidobacterium and the effectiveness of PD‐L1 antibody treatment, which involves improved dendritic cell activity [18].

Recent research has emphasized the microbiota's pivotal involvement in the response to cancer immunotherapy. Immunotherapeutic response may be adversely impacted by intestinal dysbiosis, whether as a result of disease or antibiotic usage [83]. Dysbiosis may negatively affect immunotherapy because the gut microbiota modulates Pattern recognition receptors (PRRs) which in turn affect the local and systemic antitumor immune response. Infectious strains of *Clostridium difficile*, vancomycin‐resistant enterococci (VRE), extended‐spectrum beta‐lactamase (ESBL) organisms, carbapenem‐resistant enterococci (CRE) bacteria, and Candida species can also be preferentially selected in the gut by dysbiosis. The results of therapy and survival may be significantly impacted by these organisms. Alternatively, antibiotics can enhance the mucosal immune response to immunotherapy and semi‐selectively treat dysbiosis that may have initially contributed to the cancer. The addition of gut commensals reduces tumor burden and enhances immunotherapy. Mice with melanoma were given oral gavage of *B. fragilis*, which induced a particular T‐cell response and improved the effectiveness of CTLA‐4 inhibition [84].

The intestinal microbiota is the primary subject of current research, however there are significant differences between the respiratory and intestinal microbiota, including differences in composition and function. More study is needed to understand how the intestinal and respiratory microbiomes differ in their roles in tumor therapy, particularly with regard to the impact of alterations in the local microenvironment on lung cancer patients receiving medication.

## 6 Conclusion and Future Prospective

Before any future consideration about microbiome and lung cancer, some points need to be addressed; which include (1) The evidence for the association of lung microbiome (or microbiota) and lung cancer development and progression, (2) the key microbiota that contribute to lung cancer, (3) Pathogenesis of these microbiome alteration, (4) non‐microbiota parts of the microbiome and lung cancer. In the current study, we tried to provide an overview of each one of these aspects.

The human and oral microbiomes, characterized by their varied microbial populations, play crucial roles in modulating host functions. Convincing data from studies on both humans and animals indicates that the microbiota might play a role in cancer initiation, influenced by internal and environmental factors of the host. Notably, the lung harbors its own microbiome, as do lung cancers. Despite advancements in omics technologies facilitating the study of the lung cancer microbiome, caution must be exercised in experiment conduct and interpretation due to potential contaminants and challenges in establishing causation. Nevertheless, promising initial data underscores the importance of a thorough investigation into the lung cancer microbiome to explore its potential for enhancing therapeutic strategies against this lethal disease.

In general view, it seems microbiome diversity in lung cancer patients is reduced. Meanwhile, some genera were increased in lung cancer patients in comparison with a noncancerous population (such as *Streptococcus* genus), and some of them were decreased (*Granulicatella adiacens*, *G. adiacens*).

A comprehensive overview of the microbiome's involvement in carcinogenesis, particularly its association with lung cancer, has been provided. Advanced sequencing techniques have confirmed the existence of the microbiome in the lower airways, challenging previous assumptions. The pathological significance of this microbiome varies based on host factors, lung biology, and microbial exposure. There is considerable evidence backing the likelihood of this connection, indicating potential therapeutic implications. However, the efficacy of microbiome modification for either preventing carcinogenesis or treating cancer has yet to be conclusively demonstrated. Urgent attention is needed to address challenges such as specimen selection, DNA extraction methods, and analysis technologies. Given that research on the microbiome‐carcinogenesis relationship is still in its infancy, much remains to be fully understood. Large‐scale studies in the future are expected to accumulate new insights into the prevention and treatment of lung cancer. More information about the diversity and frequency of microbiome is required to provide a clear conclusion about microbiome and pathogenesis of malignancies, especially lung cancer. More comprehensive studies with more sample sizes and high throughput methods are critical for enlightening the microbiome science in lung cancer development and possible future use in therapeutic approaches. By considering our current understanding we can suggest more comprehensive primary studies about the microbiome in lung cancer patients with more sample size in case‐control settings to provide more understanding about the pathogenesis of lung cancer. This will be a start for future possible therapeutic applications in this field.

## Author Contributions

**Reza Emadi:** conceptualization, investigation, visualization, writing–review and editing. **Sasan Saki:** conceptualization, writing–original draft, writing–review and editing. **Parastoo Yousefi:** conceptualization, data curation, writing–original draft, writing–review and editing. **Alireza Tabibzadeh:** conceptualization, data curation, project administration, writing–original draft, writing–review and editing.

## Conflicts of Interest

The authors declare no conflicts of interest.

## Data Availability

The authors have nothing to report.
